# Supplementary material for: B cell maturation antigen (BCMA) is dispensable for the survival of long-lived plasma cells
Source: Nat Commun. 2025 Aug 2;16:7106. doi: 10.1038/s41467-025-62530-2 (PMC12318048; doi:10.1038/s41467-025-62530-2)
Supplement: Supplementary file 1 — Supplementary Information [file 41467_2025_62530_MOESM1_ESM.pdf]

SUPPLEMENTAL MATERIAL

B cell maturation antigen (BCMA) is dispensable for the survival of long-lived plasma cells

Shannon R. Menzel [1], Edith Roth [1], Jens Wittner [1], Stefanie Brey [2], Leonie Weckwerth [1], Jana Thomas [1], Thomas H. Winkler [2], Wolfgang Schuh [1], Hans-Martin Jäck [1,*], Katharina Pracht [1,&] and Sebastian R. Schulz [1,&]

[1] Division of Molecular Immunology, Department of Medicine 3 – Rheumatology and Immunology, Friedrich-Alexander-University Erlangen-Nürnberg, Erlangen, Germany

[2] Division of Genetics, Department Biology, Nikolaus-Fiebiger-Center for Molecular Medicine, Friedrich-Alexander-University Erlangen-Nürnberg, Erlangen, Germany

[&] Equally Contributing Authors

* Corresponding author

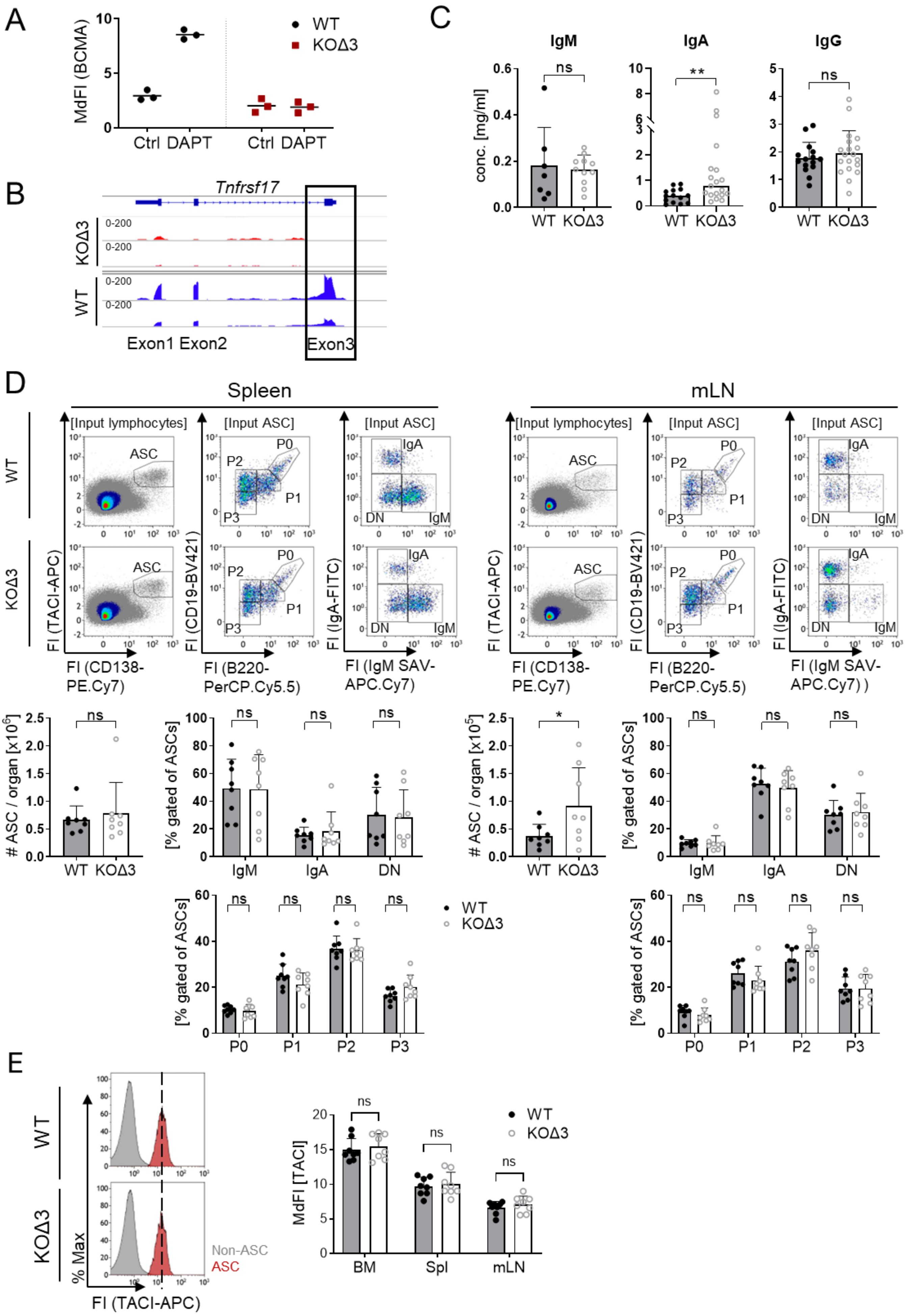
A
MdFI (BCMA)
WT
KOΔ3
Ctrl DAPT Ctrl DAPT
B
Tnfrsf17
KOΔ3
WT
0-200
Exon1 Exon2
Exon3
C
IgM
IgA
IgG
conc. [mg/ml]
ns
**
WT KOΔ3
D
Spleen
mLN
[Input lymphocytes]
[Input ASC]
ASC
P0
P1
P2
P3
IgA
DN
IgM
FI (TACI-APC)
FI (CD19-BV421)
FI (IgA-FITC)
FI (CD138-PE.Cy7)
FI (B220-PerCP.Cy5.5)
FI (IgM SAV-APC.Cy7)
ASC / organ [x10^5]
[% gated of ASCs]
*
E
% Max
FI (TACI-APC)
Non-ASC
ASC
MdFI [TACI]
BM
Spl
mLN

Supplementary Figure 1: Characterization of the BCMA KOΔ3 mice.

A Flow cytometric analysis of BCMA cell surface abundance on $CD138^+TACI^+$ ASCs using the anti-BCMA antibody clone 25C2 on splenic single-cell suspensions treated with the DAPT or solvent control (Ctrl) for 18 hours, horizontal lines indicate the mean, n=3 mice per genotype. **B** mRNA reads of BCMA-KOΔ3 (red) and wildtype (WT, blue) ASC samples were matched to the *Tnfrsf17* sequence (dark blue). The height of the peak reflects the number of aligned reads at the indicated position. The black square indicates the position of the loxP-flanked exon 3 on the genomic sequence. **C** ELISA to quantify the IgH-chain isotype-specific antibody concentrations in the serum of untreated BCMA-KOΔ3 and control mice (WT). The bar diagrams show mean and SD, with each dot indicating one mouse (n = 6 WT and 10 KOΔ3 for IgM; n = 19 per genotype for IgA and IgG). Statistical analysis was performed with two-tailed unpaired t-tests or a two-tailed Mann-Whitney U test if testing for Gaussian distribution was not passed (for serum IgA). **D** Representative gating strategy to quantify frequencies of $CD138^+TACI^+$ ASCs and ASC subpopulations P0 ($B220^{hi}CD19^{hi}$), P1 ($B220^+CD19^+$), P2 ($B220^-CD19^+$) and P3 ($B220^-CD19^-$) in spleen and mLN. ASC numbers and frequencies of the ASC subsets are shown as bar diagrams with the mean and SD (n=8 mice per group from 3 independent experiments). Statistical analysis was performed with unpaired two-tailed t-tests to compare total ASC numbers. Comparisons of ASC subsets were conducted by two-way ANOVA with Šídák's correction for multiple comparisons. **E** Flow cytometric analysis of TACI abundance on ASCs isolated from bone marrow, spleen (Spl) and mLN of untreated BCMA-KOΔ3 and wildtype mice. The median fluorescent intensity (MdFI) of ASCs is normalized to TACI MdFI of non-ASCs (lymphocytes excluding $CD138^-TACI^-$ ASC) in the same sample. Histograms show representative data from the bone marrow (BM). Comparisons of TACI abundances were conducted by two-way ANOVA with Šídák's correction for multiple comparisons with n=8 mice per group from 3 independent experiments. Exact p-values are provided in the Source Data file. ns = not significant, * = $p \leq 0.05$, ** = $p \leq 0.01$. ASC = antibody-secreting cell, DN= double negative (IgA^-IgM^-), mLN = mesenteric lymph node.

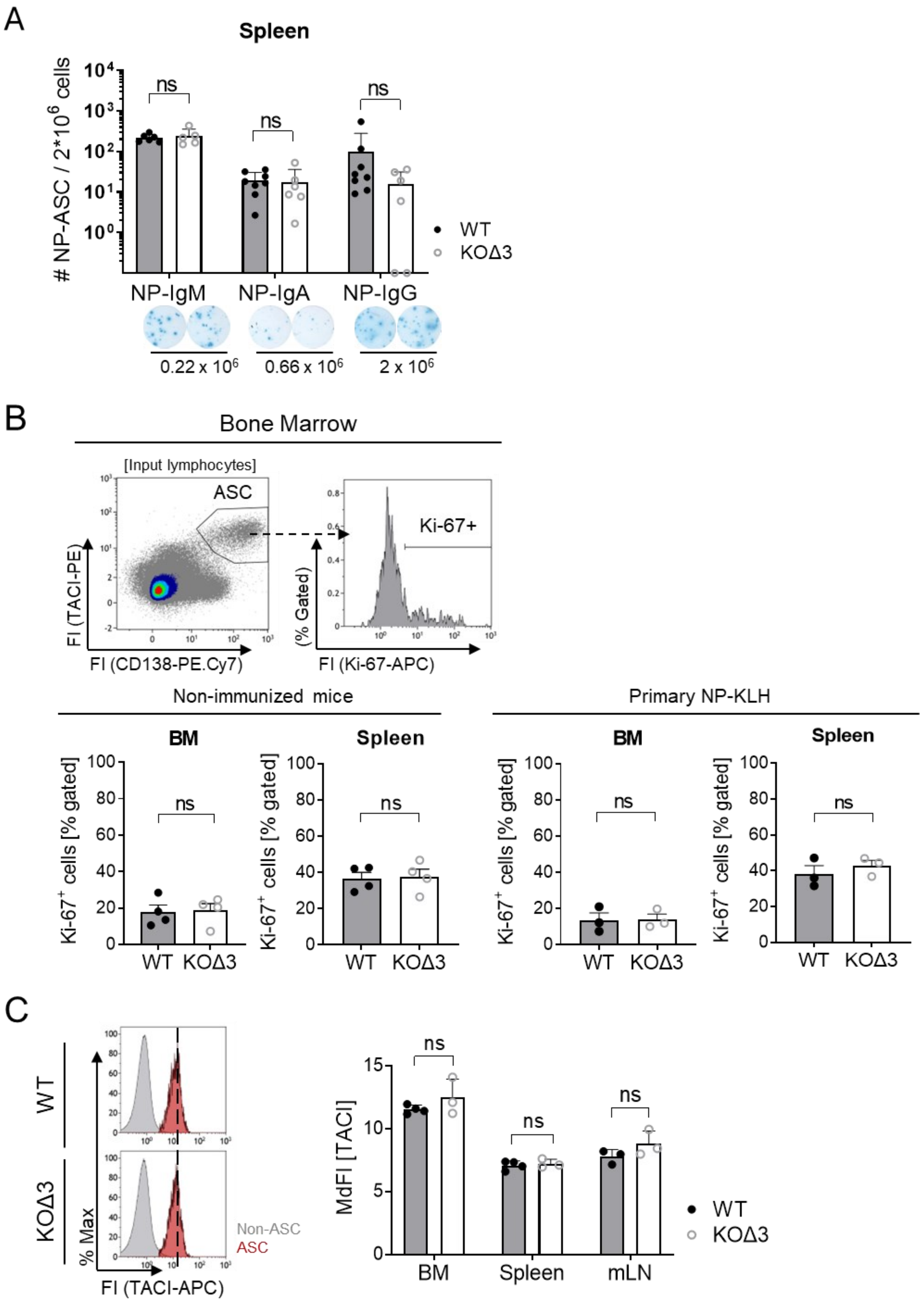
A
Spleen
NP-ASC / 2*10^6 cells
ns
ns
ns
WT
KOΔ3
NP-IgM
NP-IgA
NP-IgG
0.22 x 10^6
0.66 x 10^6
2 x 10^6
B
Bone Marrow
[Input lymphocytes]
ASC
Ki-67+
FI (TACI-PE)
FI (CD138-PE.Cy7)
(% Gated)
FI (Ki-67-APC)
Non-immunized mice
Primary NP-KLH
BM
Spleen
BM
Spleen
Ki-67+ cells [% gated]
ns
WT
KOΔ3
C
WT
KOΔ3
% Max
FI (TACI-APC)
Non-ASC
ASC
MdFI [TACI]
ns
BM
Spleen
mLN
WT
KOΔ3

Supplementary Figure 2: Characterization of the BCMA-KOΔ3 mice after primary immunization with NP-KLH in alum.

A IgH-chain isotype-specific quantification of antigen (NP)-specific ASCs by ELISpot analysis in the spleen for wildtype (WT, black) and BCMA-KOΔ3 mice (KOΔ3, grey). Images below graphs are representative pictures of ELISpot analysis with numbers indicating the number of cells seeded per well. Statistical significance between WT and KOΔ3 for each isotype was assessed by two-way ANOVA with Šídák's multiple comparisons correction (n = 6 per group for IgM, n=8 WT and 6 KOΔ3 for IgA/IgG). **B** Flow cytometric analysis of Ki-67 protein abundance in bone marrow (BM) and splenic ASCs from BCMA-KOΔ3 and wildtype mice. Mice were analyzed either unimmunized (n=4 per group) or 7 weeks after primary immunization with NP-KLH in Alum (n=3 per group). Ki-67+ ASC numbers for WT and KOΔ3 in non-immunized mice were compared within each organ by two-way ANOVA with Šídák's multiple comparisons correction. **C** Flow cytometric analysis of TACI abundance on ASCs isolated from bone marrow (BM), spleen and mesenteric lymph nodes (mLN) of WT and BCMA-KOΔ3 mice immunized with 100µg NP-KLH in alum and analyzed after 7 weeks. The median fluorescent intensity (MdFI) of TACI on ASCs is normalized to the TACI MdFI of non-ASCs (lymphocytes excluding $CD138^+TACI^+$ ASC) in the same sample. Histograms show representative data from the bone marrow. Data represent mean and SD (n = 4 mice per genotype). Statistical comparisons were made by two-way ANOVA with Šídák's correction for multiple comparisons. Exact p-values are provided in the Source Data file.

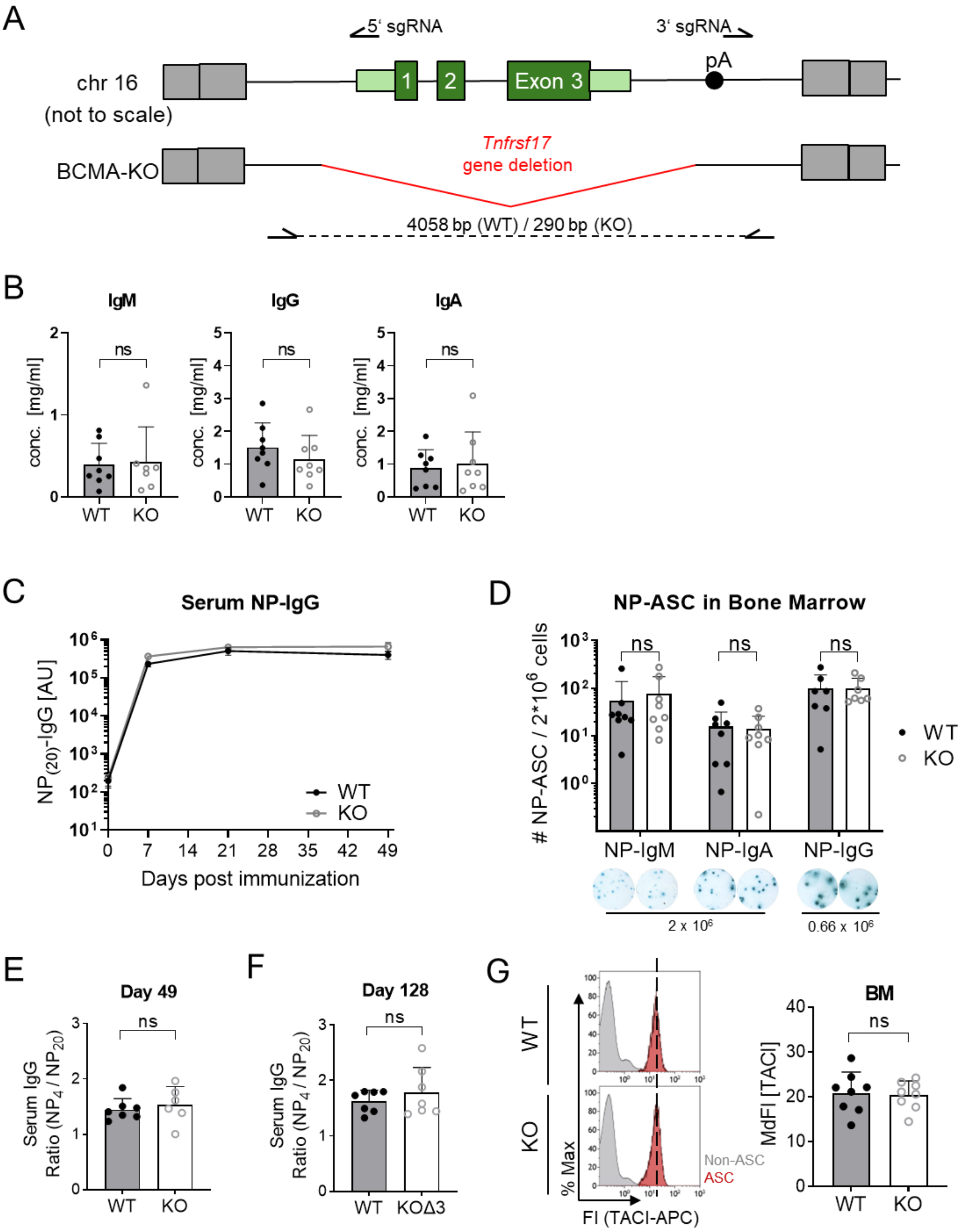

Supplementary Figure 3: Characterisation of the BCMA-deficient mice generated by genomic deletion of the complete *Tnfrsf17* locus.

A Schematic illustration of the *Tnfrsf17* gene locus on chromosome (chr) 16 with the location of the single guide (sg)RNAs for CRISPR-Cas9 mediated knock-out (BCMA-KO mice). Dark green boxes indicate the three exons of the *Tnfrsf17* gene, and light green boxes indicate the 5’ and 3’ UTR. **B** IgH-chain isotype-specific antibody serum concentrations of untreated mice were determined by ELISA for wildtype (WT, black,

n=8) and BCMA-KO mice (KO, grey, n=8). **C** NP-specific IgG serum concentrations were determined by ELISA for wildtype (black, n=7) and BCMA-KO mice (grey, n=7) after primary immunization with NP-KLH in alum. **D** IgH-chain isotype-specific quantification of antigen-specific (NP) ASCs by ELISpot analysis in bone marrow for wildtype (WT, black) and BCMA-KO mice (KOΔ3, grey). Images below graphs are representative pictures of ELISpot analysis with numbers indicating the number of cells seeded per well. Bar diagrams show mean and SD with each dot indicating one mouse (n=8 mice per genotype for NP-IgM/NP-IgA, n=7 mice per genotype for NP-IgG). **E, F** Binding affinities of serum NP-IgG assessed by the ratio of $NP_{(4)}/NP_{(20)}$ of wildtype mice (WT) and BCMA-KO mice (KO) at day 49 after immunization with 100 µg NP-KLH in alum (E) and of wildtype mice (WT) and BCMA-KOΔ3 mice (KOΔ3) at day 128 after immunization with 100 µg NP-KLH in alum and boost with 50 µg NP-KLH in PBS (F). Data represent mean and SD (n = 7 mice per genotype). **G** Flow cytometric analysis of TACI abundance on bone marrow ASCs isolated from BCMA-KO immunized with 100 µg NP-KLH in alum and analyzed after 7 weeks. The median fluorescent intensity (MdFI) of ASCs is normalized to TACI MdFI of non-ASCs (lymphocytes excluding $CD138^{+}TACI^{+}$) in the same sample. Data represent mean and SD (n = 8 WT mice and 8 BCMA-KO mice). Statistical analysis in B-D was performed using a two-way ANOVA with Šídák's multiple comparisons test. Comparisons in E-G were performed with two-tailed unpaired t-tests, correcting for multiple comparisons by the False Discovery Rate (FDR) according to Benjamini, Krieger and Yekutieli Two-stage step-up Method (E,F). Exact p-values are described in the Source Data file. ns = not significant, pA = polyA site, ASC = antibody-secreting cell.

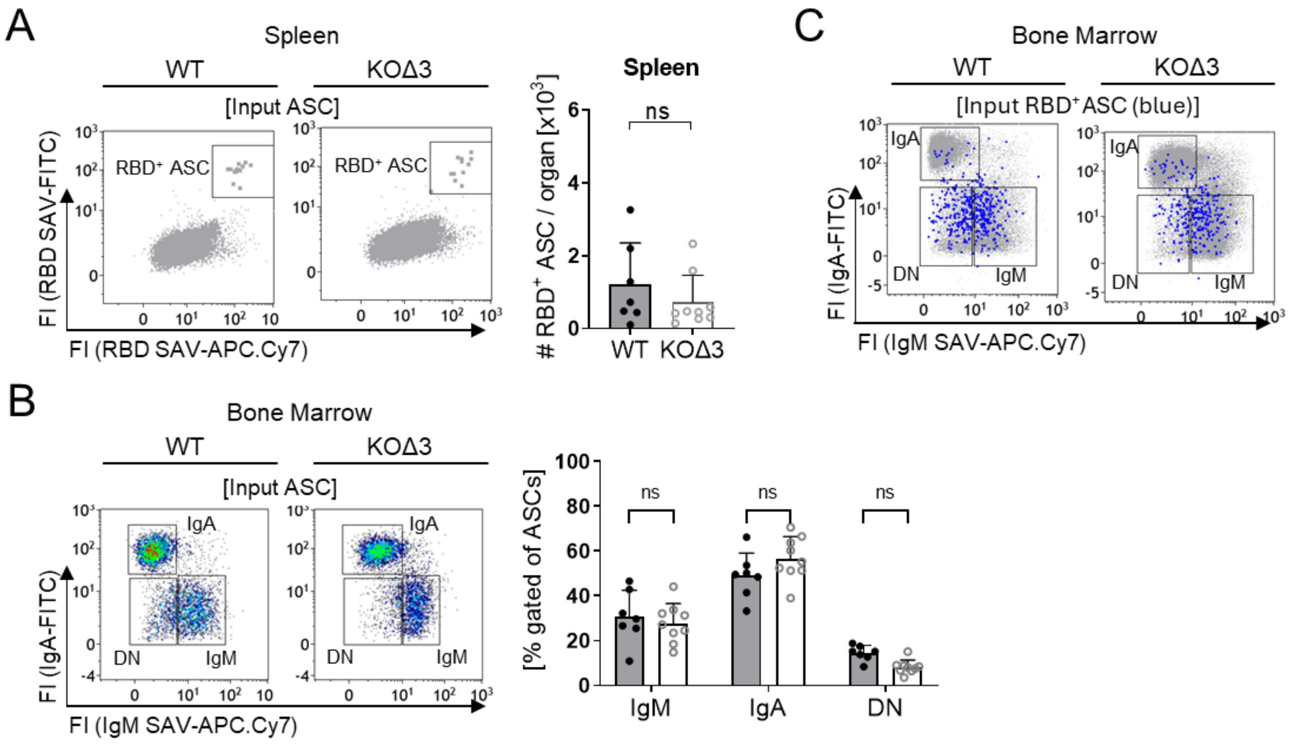

Supplementary Figure 4: Flow cytometric analysis of total and antigen-specific ASCs in BCMA-KOΔ3 and wildtype mice upon prime/boost immunization with the mRNA vaccine mRNA-1273.

Wildtype (WT) and BCMA-KOΔ3 mice were immunized with mRNA-1273 and boosted on day42. Representative gating to quantify **A** RBD-specific $CD138^{+}TACI^{+}$ ASC numbers in the spleen by intracellular staining of RBD, **B** the frequency of IgH-chain isotype-specific bone marrow ASCs and **C** the IgH-chain isotype-specificity of antigen-specific ASCs. Displayed in grey are the events of all ASCs, and antigen-specific ASCs are labeled in blue. Analyses were performed on day 126 after primary immunization with mRNA-1273. Bars represent mean and SD (n = 7 WT mice and 8 BCMA-KOΔ3 mice). Statistical comparisons were made by two-tailed unpaired t-test (A) and two-way ANOVA with Šídák's correction for multiple comparisons (B). Exact p-values are described in the Source Data file. ns = not significant, * = $p \leq 0.05$. ASC = antibody-secreting cells, DN= double negative ($IgA^{-}IgM^{-}$), RBD = receptor binding domain of SARS-CoV-2.

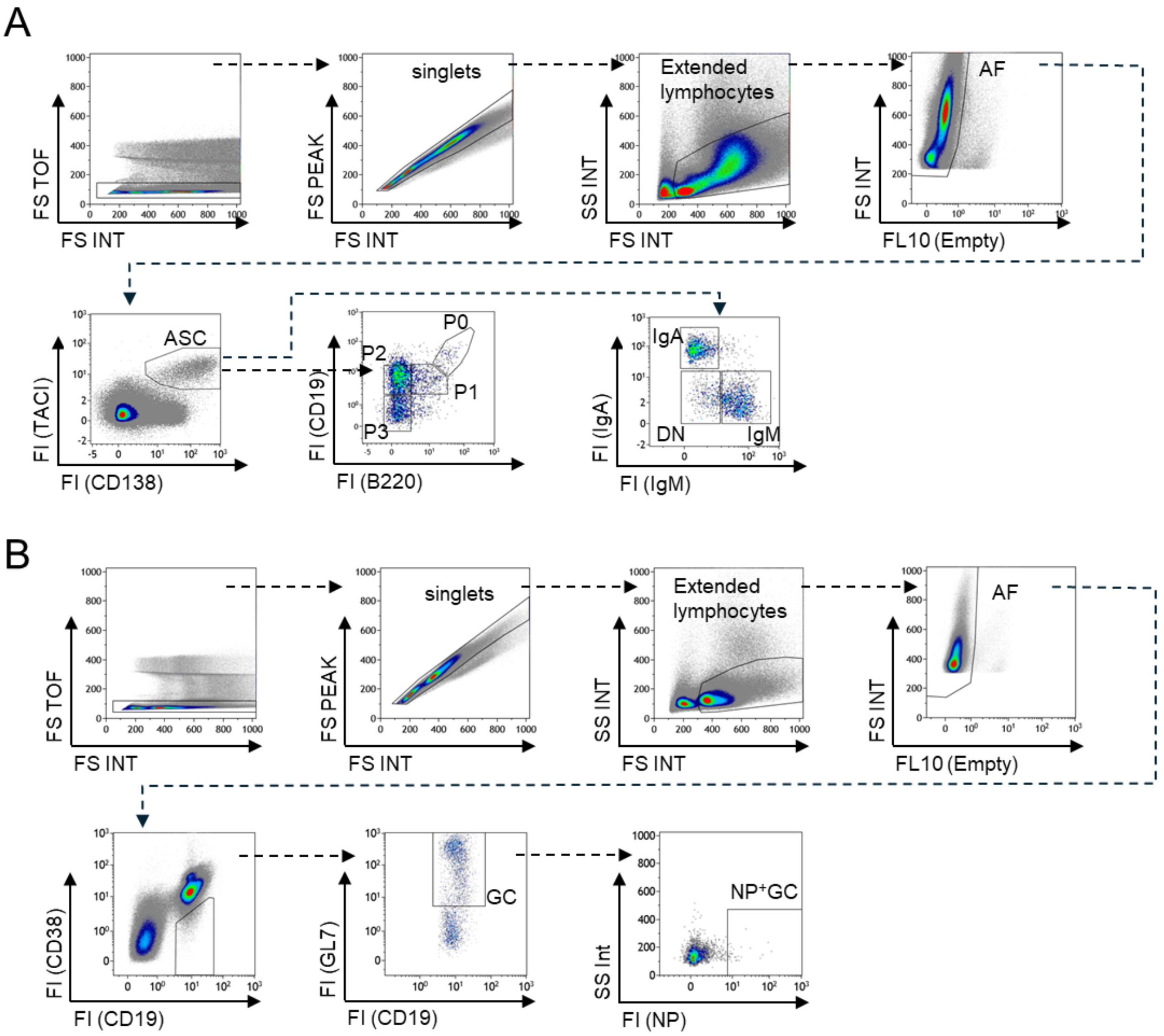

Supplementary Figure 5: Gating strategy for flow cytometric analysis of B cell and plasma cell populations.

Representative gating strategy to quantify **A** antibody-secreting cell populations and **B** antigen (NP)-specific germinal center B cell populations. Extended lymphocytes excluding autofluorescence by the empty channel FL10 were used as input for all plots gating on $CD138^{+}TACI^{+}$ ASCs as indicated (Fig.1B,C, Fig.2D, Fig.3D, Fig.4C,D, Fig. S1D. Fig. S2B, Fig. S4B,C) and for $CD19^{+}CD38^{lo}$ B cells (Fig. 2E). AF = autofluorescence, ASC = antibody-secreting cell, DN= double negative ($IgA^{-}IgM^{-}$), GC = germinal center.
