# Supplementary material for: B cell maturation antigen (BCMA) is dispensable for the survival of long-lived plasma cells
Source: Nat Commun. 2025 Aug 2;16:7106. doi: 10.1038/s41467-025-62530-2 (PMC12318048; doi:10.1038/s41467-025-62530-2)
Supplement: Supplementary file 2 — Reporting Summary [file 41467_2025_62530_MOESM2_ESM.pdf]

nature portfolio

Corresponding author(s): Hans-Martin Jäck

Last updated by author(s): Jun 30, 2025

Reporting Summary

Nature Portfolio wishes to improve the reproducibility of the work that we publish. This form provides structure for consistency and transparency in reporting. For further information on Nature Portfolio policies, see our Editorial Policies and the Editorial Policy Checklist.

Statistics

For all statistical analyses, confirm that the following items are present in the figure legend, table legend, main text, or Methods section.

n/a	Confirmed	
☐	☒	The exact sample size (*n*) for each experimental group/condition, given as a discrete number and unit of measurement
☐	☒	A statement on whether measurements were taken from distinct samples or whether the same sample was measured repeatedly
☐	☒	The statistical test(s) used AND whether they are one- or two-sided<br>*Only common tests should be described solely by name; describe more complex techniques in the Methods section.*
☒	☐	A description of all covariates tested
☐	☒	A description of any assumptions or corrections, such as tests of normality and adjustment for multiple comparisons
☐	☒	A full description of the statistical parameters including central tendency (e.g. means) or other basic estimates (e.g. regression coefficient) AND variation (e.g. standard deviation) or associated estimates of uncertainty (e.g. confidence intervals)
☐	☒	For null hypothesis testing, the test statistic (e.g. *F*, *t*, *r*) with confidence intervals, effect sizes, degrees of freedom and *P* value noted<br>*Give P values as exact values whenever suitable.*
☒	☐	For Bayesian analysis, information on the choice of priors and Markov chain Monte Carlo settings
☒	☐	For hierarchical and complex designs, identification of the appropriate level for tests and full reporting of outcomes
☒	☐	Estimates of effect sizes (e.g. Cohen's *d*, Pearson's *r*), indicating how they were calculated

Our web collection on statistics for biologists contains articles on many of the points above.

Software and code

Policy information about availability of computer code

Data collection	Gallios Flow Cytometer (Beckman Coulter), MoFlo Astrios Cell Sorter (Beckman Coulter), Mini ELISA Plate Reader (BioLegend), ImmunoSpot S6 Ultimate ELISpot Reader (CLS)
Data analysis	Graphpad Prism (v9.4); Kaluza (v2.2); R (v4.3); edgeR (v4.0.0); tidyverse (v2.0.0); STAR (v2.7.10); salmon (v1.10.01); ImmunoSpot (v5.1.36)

For manuscripts utilizing custom algorithms or software that are central to the research but not yet described in published literature, software must be made available to editors and reviewers. We strongly encourage code deposition in a community repository (e.g. GitHub). See the Nature Portfolio guidelines for submitting code & software for further information.

Data

Policy information about availability of data

All manuscripts must include a data availability statement. This statement should provide the following information, where applicable:

- Accession codes, unique identifiers, or web links for publicly available datasets
- A description of any restrictions on data availability
- For clinical datasets or third party data, please ensure that the statement adheres to our policy

The RNA-seq data generated in this study are available in the NCBI Gene Expression Omnibus (GEO) database under accession number GSE277098. All other data are provided within the article and Supplementary Materials.

Research involving human participants, their data, or biological material

Policy information about studies with human participants or human data. See also policy information about sex, gender (identity/presentation), and sexual orientation and race, ethnicity and racism.

Reporting on sex and gender	*Use the terms sex (biological attribute) and gender (shaped by social and cultural circumstances) carefully in order to avoid confusing both terms. Indicate if findings apply to only one sex or gender; describe whether sex and gender were considered in study design; whether sex and/or gender was determined based on self-reporting or assigned and methods used. Provide in the source data disaggregated sex and gender data, where this information has been collected, and if consent has been obtained for sharing of individual-level data; provide overall numbers in this Reporting Summary. Please state if this information has not been collected. Report sex- and gender-based analyses where performed, justify reasons for lack of sex- and gender-based analysis.*
Reporting on race, ethnicity, or other socially relevant groupings	*Please specify the socially constructed or socially relevant categorization variable(s) used in your manuscript and explain why they were used. Please note that such variables should not be used as proxies for other socially constructed/relevant variables (for example, race or ethnicity should not be used as a proxy for socioeconomic status). Provide clear definitions of the relevant terms used, how they were provided (by the participants/respondents, the researchers, or third parties), and the method(s) used to classify people into the different categories (e.g. self-report, census or administrative data, social media data, etc.) Please provide details about how you controlled for confounding variables in your analyses.*
Population characteristics	*Describe the covariate-relevant population characteristics of the human research participants (e.g. age, genotypic information, past and current diagnosis and treatment categories). If you filled out the behavioural & social sciences study design questions and have nothing to add here, write "See above."*
Recruitment	*Describe how participants were recruited. Outline any potential self-selection bias or other biases that may be present and how these are likely to impact results.*
Ethics oversight	*Identify the organization(s) that approved the study protocol.*

Note that full information on the approval of the study protocol must also be provided in the manuscript.

Field-specific reporting

Please select the one below that is the best fit for your research. If you are not sure, read the appropriate sections before making your selection.

☒ Life sciences ☐ Behavioural & social sciences ☐ Ecological, evolutionary & environmental sciences

For a reference copy of the document with all sections, see nature.com/documents/nr-reporting-summary-flat.pdf

Life sciences study design

All studies must disclose on these points even when the disclosure is negative.

Sample size	Sample sizes were not predetermined by formal statistical calculation but were chosen based on our prior experience with similar assays and practical feasibility. Specific details about the sample sizes and the number of independent experiments are described in the corresponding figure legends.
Data exclusions	Only data from animals and samples meeting predefined quality-control criteria (correct genotype, no signs of infection or inflammation, successful control staining or gene detection, and a bona fide antibody response by day 14 post-immunization) were included in the analysis. Mice failing to mount an antigen-specific IgG response at day 14 were excluded.<br>In detail:<br>Fig. 2B,C,E: 4 KO mice were excluded, as they failed to mount an antigen-specific IgG response.<br>Fig. 3B,C: 3 KO mice were excluded, as they failed to mount an antigen-specific IgG response.<br>Fig. 4B,C: 1 WT mouse was excluded, as it failed to mount an antigen-specific IgG response.<br>Further, statistical outlier were identified by Grubb's outlier test and a maximum of 1 outlier per group was excluded.
Replication	All experiments were conducted at least twice independently. Detailed information regarding sample sizes and numbers of independent experiments is provided in the figure legends. All experiments were reliably reproduced.
Randomization	Animals were assigned into experimental groups based on their genotypes. Experiments were conducted with sex- and age matching between treatment groups. Animals were littermates or co-housed for at least 2 weeks before each experiment.
Blinding	Blinding was not performed, as investigators oversaw all steps from genotype confirmation to analysis, although flow cytometry sample acquisition was conducted without knowledge of genotype.

Behavioural & social sciences study design

All studies must disclose on these points even when the disclosure is negative.

Study description	*Briefly describe the study type including whether data are quantitative, qualitative, or mixed-methods (e.g. qualitative cross-sectional, quantitative experimental, mixed-methods case study).*
Research sample	*State the research sample (e.g. Harvard university undergraduates, villagers in rural India) and provide relevant demographic information (e.g. age, sex) and indicate whether the sample is representative. Provide a rationale for the study sample chosen. For studies involving existing datasets, please describe the dataset and source.*
Sampling strategy	*Describe the sampling procedure (e.g. random, snowball, stratified, convenience). Describe the statistical methods that were used to predetermine sample size OR if no sample-size calculation was performed, describe how sample sizes were chosen and provide a rationale for why these sample sizes are sufficient. For qualitative data, please indicate whether data saturation was considered, and what criteria were used to decide that no further sampling was needed.*
Data collection	*Provide details about the data collection procedure, including the instruments or devices used to record the data (e.g. pen and paper, computer, eye tracker, video or audio equipment) whether anyone was present besides the participant(s) and the researcher, and whether the researcher was blind to experimental condition and/or the study hypothesis during data collection.*
Timing	*Indicate the start and stop dates of data collection. If there is a gap between collection periods, state the dates for each sample cohort.*
Data exclusions	*If no data were excluded from the analyses, state so OR if data were excluded, provide the exact number of exclusions and the rationale behind them, indicating whether exclusion criteria were pre-established.*
Non-participation	*State how many participants dropped out/declined participation and the reason(s) given OR provide response rate OR state that no participants dropped out/declined participation.*
Randomization	*If participants were not allocated into experimental groups, state so OR describe how participants were allocated to groups, and if allocation was not random, describe how covariates were controlled.*

Ecological, evolutionary & environmental sciences study design

All studies must disclose on these points even when the disclosure is negative.

Study description	*Briefly describe the study. For quantitative data include treatment factors and interactions, design structure (e.g. factorial, nested, hierarchical), nature and number of experimental units and replicates.*
Research sample	*Describe the research sample (e.g. a group of tagged Passer domesticus, all Stenocereus thurberi within Organ Pipe Cactus National Monument), and provide a rationale for the sample choice. When relevant, describe the organism taxa, source, sex, age range and any manipulations. State what population the sample is meant to represent when applicable. For studies involving existing datasets, describe the data and its source.*
Sampling strategy	*Note the sampling procedure. Describe the statistical methods that were used to predetermine sample size OR if no sample-size calculation was performed, describe how sample sizes were chosen and provide a rationale for why these sample sizes are sufficient.*
Data collection	*Describe the data collection procedure, including who recorded the data and how.*
Timing and spatial scale	*Indicate the start and stop dates of data collection, noting the frequency and periodicity of sampling and providing a rationale for these choices. If there is a gap between collection periods, state the dates for each sample cohort. Specify the spatial scale from which the data are taken*
Data exclusions	*If no data were excluded from the analyses, state so OR if data were excluded, describe the exclusions and the rationale behind them, indicating whether exclusion criteria were pre-established.*
Reproducibility	*Describe the measures taken to verify the reproducibility of experimental findings. For each experiment, note whether any attempts to repeat the experiment failed OR state that all attempts to repeat the experiment were successful.*
Randomization	*Describe how samples/organisms/participants were allocated into groups. If allocation was not random, describe how covariates were controlled. If this is not relevant to your study, explain why.*
Blinding	*Describe the extent of blinding used during data acquisition and analysis. If blinding was not possible, describe why OR explain why blinding was not relevant to your study.*

Did the study involve field work? ☐ Yes ☒ No

Field work, collection and transport

Field conditions	*Describe the study conditions for field work, providing relevant parameters (e.g. temperature, rainfall).*
Location	*State the location of the sampling or experiment, providing relevant parameters (e.g. latitude and longitude, elevation, water depth).*
Access & import/export	*Describe the efforts you have made to access habitats and to collect and import/export your samples in a responsible manner and in compliance with local, national and international laws, noting any permits that were obtained (give the name of the issuing authority, the date of issue, and any identifying information).*
Disturbance	*Describe any disturbance caused by the study and how it was minimized.*

Reporting for specific materials, systems and methods

We require information from authors about some types of materials, experimental systems and methods used in many studies. Here, indicate whether each material, system or method listed is relevant to your study. If you are not sure if a list item applies to your research, read the appropriate section before selecting a response.

Materials & experimental systems

n/a	Involved in the study	
☐	☒	Antibodies
☒	☐	Eukaryotic cell lines
☒	☐	Palaeontology and archaeology
☐	☒	Animals and other organisms
☒	☐	Clinical data
☒	☐	Dual use research of concern
☒	☐	Plants

Methods

n/a	Involved in the study	
☒	☐	ChIP-seq
☐	☒	Flow cytometry
☒	☐	MRI-based neuroimaging

Antibodies

Antibodies used

Recombinant antibody against human/mouse BCMA (clone 25C2) was produced and purified in-house from HEK293 cell supernatant as described in Schulz et al. (10.3389/fimmu.2025.1539773).

The following antibodies were used for flow cytometry and purchased from commercial suppliers:
anti-mouse CD138-PE.Cy7 (clone 281-2, biolegend, Cat.: 142514, 1:1500)
anti-mouse TACI-APC (clone eBio8F10-3, ebioscience, Cat.: 17-5942, 1:400)
anti-mouse TACI-BV421 (clone 8F10, BD, Cat.: 742840, 1:600)
anti-mouse B220-PerCPCy5.5 (clone Ra3-6b2, ebioscience, Cat.: 45-0452-80, 1:200)
anti-mouse CD19-BV421 (clone 6D5, biolegend, Cat.: 115538, 1:200)
anti-mouse IgA-FITC (polyclonal, Southern Biotech, Cat.: 1040-02, 1:1000)
anti-mouse IgA-AF647 (polyclonal, Southern Biotech, Cat.: 1040-31, 1:10000)
anti-mouse IgM-Biotin (polyclonal, Jackson, Cat.: 115-065-075, 1:1000)
anti-human IgG-AF647 (polyclonal, Southern Biotech, Cat.: 2048-31, 1:1000)
anti-mouse CD38-APC.Cy7 (clone 90, biolegend, Cat.: 102727, 1:1000)
anti-mouse GL7-FITC (clone GL7, BD, Cat.: 562080, 1:100)
NP(28)-PE (Biosearch, Cat.: N-5070-1, 1:200)
Sav-FITC (ebioscience, Cat.: 11-4317-87, 1:500)
Sav-APC.Cy7 (biolegend, Cat.: 405208, 1:1600)
anti-mouse CD19-APCFire750 (clone 6D5, biolegend, Cat.: 115558, 1:400)
anti-mouse CD38-PerCPCy5.5 (clone 90, biolegend, Cat.: 102722, 1:100)
anti-mouse CD16/32- (clone 93, ThermoFisher, Cat.: 14-0161-82, 1:100)

The following polyclonal antibodies were used for ELISA and ELISpot assays and purchased from commercial suppliers:
anti-mouse IgM (Southern Biotech, Cat.: 1021-01)
anti-mouse IgG (Southern Biotech, Cat.: 1030-01)
anti-mouse IgA (Southern Biotech, Cat.: 1040-01)
anti-mouse IgM-HRP (Southern Biotech Cat.: 1021-05)
anti-mouse IgG-HRP (Southern Biotech Cat.: 1030-05)
anti-mouse IgA-HRP (Southern Biotech Cat.: 1040-05)
anti-mouse IgM-AP (Southern Biotech Cat.: 1021-04)
anti-mouse IgG-AP (Southern Biotech Cat.: 1030-04)
anti-mouse IgA-AP (Southern Biotech Cat.: 1040-04)

Validation

Validity of in-house produced anti-BCMA antibody was confirmed by flow cytometry using a plasma cell staining of bone marrow

Validation	from wildtype and BCMA-deficient mice as well as isotype-matched control antibodies (Fig. 1) and further validated in HEK cells transfected with a control vector or a vector encoding mouse BCMA. All other antibodies obtained from commercial sources are well-established and were validated by the respective manufacturer. For detailed information, please refer to the respective product page using the provided clone or catalogue number. All antibodies were titrated in-house for optimal performance.

Palaeontology and Archaeology

Specimen provenance	*Provide provenance information for specimens and describe permits that were obtained for the work (including the name of the issuing authority, the date of issue, and any identifying information). Permits should encompass collection and, where applicable, export.*
Specimen deposition	*Indicate where the specimens have been deposited to permit free access by other researchers.*
Dating methods	*If new dates are provided, describe how they were obtained (e.g. collection, storage, sample pretreatment and measurement), where they were obtained (i.e. lab name), the calibration program and the protocol for quality assurance OR state that no new dates are provided.*

☐ Tick this box to confirm that the raw and calibrated dates are available in the paper or in Supplementary Information.

Ethics oversight	*Identify the organization(s) that approved or provided guidance on the study protocol, OR state that no ethical approval or guidance was required and explain why not.*

Note that full information on the approval of the study protocol must also be provided in the manuscript.

Animals and other research organisms

Policy information about studies involving animals; ARRIVE guidelines recommended for reporting animal research, and Sex and Gender in Research

Laboratory animals	BCMA-KO mice were generated in this study as described using CRISPR/Cas9 in C57BL/6 zygotes. BCMA_KO3 mice were generated by crossing BCMA:Tom mice with E2A-Cre deleter mice for the genomic deletion of BCMA Exon 3.<br>C57BL/6NRj wild-type mice were purchased from Janvier (Le Genest Saint Isle, France).<br>All mice were housed in a specific-pathogen-free facility at temperatures between 22 and 23°C , a humidity of 50%-60% and a regulated 12-hour light/dark cycle with free access to normal chow diet and water.
Wild animals	The study did not involve wild animals.
Reporting on sex	All experiments were performed with both male and female mice, with sex-matching between experimental groups.
Field-collected samples	This study did not involve samples collected from the field.
Ethics oversight	All animal experiments were performed according to institutional and national guidelines and were approved by the State of Bavaria (Amt für Veterinärwesen und gesundheitlichen Verbraucherschutz der Stadt Erlangen, Regierung von Unterfranken, Würzburg, Germany)

Note that full information on the approval of the study protocol must also be provided in the manuscript.

Clinical data

Policy information about clinical studies

All manuscripts should comply with the ICMJE guidelines for publication of clinical research and a completed CONSORT checklist must be included with all submissions.

Clinical trial registration	*Provide the trial registration number from ClinicalTrials.gov or an equivalent agency.*
Study protocol	*Note where the full trial protocol can be accessed OR if not available, explain why.*
Data collection	*Describe the settings and locales of data collection, noting the time periods of recruitment and data collection.*
Outcomes	*Describe how you pre-defined primary and secondary outcome measures and how you assessed these measures.*

Dual use research of concern

Policy information about dual use research of concern

Hazards

Could the accidental, deliberate or reckless misuse of agents or technologies generated in the work, or the application of information presented in the manuscript, pose a threat to:

No	Yes	
☒	☐	Public health
☒	☐	National security
☒	☐	Crops and/or livestock
☒	☐	Ecosystems
☒	☐	Any other significant area

Experiments of concern

Does the work involve any of these experiments of concern:

No	Yes	
☒	☐	Demonstrate how to render a vaccine ineffective
☒	☐	Confer resistance to therapeutically useful antibiotics or antiviral agents
☒	☐	Enhance the virulence of a pathogen or render a nonpathogen virulent
☒	☐	Increase transmissibility of a pathogen
☒	☐	Alter the host range of a pathogen
☒	☐	Enable evasion of diagnostic/detection modalities
☒	☐	Enable the weaponization of a biological agent or toxin
☒	☐	Any other potentially harmful combination of experiments and agents

Plants

Seed stocks

Report on the source of all seed stocks or other plant material used. If applicable, state the seed stock centre and catalogue number. If plant specimens were collected from the field, describe the collection location, date and sampling procedures.

Novel plant genotypes

Describe the methods by which all novel plant genotypes were produced. This includes those generated by transgenic approaches, gene editing, chemical/radiation-based mutagenesis and hybridization. For transgenic lines, describe the transformation method, the number of independent lines analyzed and the generation upon which experiments were performed. For gene-edited lines, describe the editor used, the endogenous sequence targeted for editing, the targeting guide RNA sequence (if applicable) and how the editor was applied.

Authentication

Describe any authentication procedures for each seed stock used or novel genotype generated. Describe any experiments used to assess the effect of a mutation and, where applicable, how potential secondary effects (e.g. second site T-DNA insertions, mosiacism, off-target gene editing) were examined.

ChIP-seq

Data deposition

☐ Confirm that both raw and final processed data have been deposited in a public database such as GEO.

☐ Confirm that you have deposited or provided access to graph files (e.g. BED files) for the called peaks.

Data access links
May remain private before publication.

For "Initial submission" or "Revised version" documents, provide reviewer access links. For your "Final submission" document, provide a link to the deposited data.

Files in database submission

Provide a list of all files available in the database submission.

Genome browser session
(e.g. UCSC)

Provide a link to an anonymized genome browser session for "Initial submission" and "Revised version" documents only, to enable peer review. Write "no longer applicable" for "Final submission" documents.

Methodology

Replicates

Describe the experimental replicates, specifying number, type and replicate agreement.

Sequencing depth

Describe the sequencing depth for each experiment, providing the total number of reads, uniquely mapped reads, length of reads and whether they were paired- or single-end.

Antibodies

Describe the antibodies used for the ChIP-seq experiments; as applicable, provide supplier name, catalog number, clone name, and lot number.

Peak calling parameters	*Specify the command line program and parameters used for read mapping and peak calling, including the ChIP, control and index files used.*
Data quality	*Describe the methods used to ensure data quality in full detail, including how many peaks are at FDR 5% and above 5-fold enrichment.*
Software	*Describe the software used to collect and analyze the ChIP-seq data. For custom code that has been deposited into a community repository, provide accession details.*

Flow Cytometry

Plots

Confirm that:

☒ The axis labels state the marker and fluorochrome used (e.g. CD4-FITC).

☒ The axis scales are clearly visible. Include numbers along axes only for bottom left plot of group (a 'group' is an analysis of identical markers).

☐ All plots are contour plots with outliers or pseudocolor plots.

☒ A numerical value for number of cells or percentage (with statistics) is provided.

Methodology

Sample preparation	Bones (femurs, tibiae) were isolated, cleaned from surrounding tissue, decapped and flushed with 27G cannula and PBS + 2% FCS. The bone marrow suspension was subsequently centrifuged at 300 × g for 5 min at 4°C. The pellet was resuspended in RBC-lysis buffer and incubated for 6 min at room temperature. Lysis was stopped by adding PBS + 2% FCS, and the cell suspension was filtered through a 30 µm mesh filter.<br>Spleens and mesenteric lymph nodes were isolated, cleaned from surrounding tissue, and gently disrupted in PBS + 2% FCS through a 70µm cell strainer using the plunger of a 2 ml syringe. The cells were pelleted by centrifugation at 300 × g for 5 min at 4°C, resuspended in RBC-Lysis buffer and incubated for 6 min at room temperature. The lysis was stopped by adding PBS + 2% FCS and the cell suspension was filtered through a 30µm mesh filter.<br>Before flow cytometric stainings, single cell suspensions were incubated with Fc-Block (anti-mouse CD16/32, clone 93, ThermoFisher) for 5 min at RT. Cell suspensions were surface stained with antibodies diluted in PBS + 2% FCS on ice for 20 min in the dark.
Instrument	Samples were acquired with a Gallios flow cytometer (Beckman Coulter)
Software	Kaluza V2.2 (Beckman Coulter)
Cell population abundance	For RNA-Seq analyses, plasma cells were sorted directly into lysis buffer, precluding post-sort purity analysis.
Gating strategy	Live cells and single cells were gated based on FSC/SSC characteristics. gating of individual B cell subsets were indicated in the respective figures.

☒ Tick this box to confirm that a figure exemplifying the gating strategy is provided in the Supplementary Information.

Magnetic resonance imaging

Experimental design

Design type	*Indicate task or resting state; event-related or block design.*
Design specifications	*Specify the number of blocks, trials or experimental units per session and/or subject, and specify the length of each trial or block (if trials are blocked) and interval between trials.*
Behavioral performance measures	*State number and/or type of variables recorded (e.g. correct button press, response time) and what statistics were used to establish that the subjects were performing the task as expected (e.g. mean, range, and/or standard deviation across subjects).*

Acquisition

Imaging type(s)	*Specify: functional, structural, diffusion, perfusion.*
Field strength	*Specify in Tesla*
Sequence & imaging parameters	*Specify the pulse sequence type (gradient echo, spin echo, etc.), imaging type (EPI, spiral, etc.), field of view, matrix size, slice thickness, orientation and TE/TR/flip angle.*
Area of acquisition	*State whether a whole brain scan was used OR define the area of acquisition, describing how the region was determined.*

Diffusion MRI ☐ Used ☐ Not used

Preprocessing

Preprocessing software — *Provide detail on software version and revision number and on specific parameters (model/functions, brain extraction, segmentation, smoothing kernel size, etc.).*

Normalization — *If data were normalized/standardized, describe the approach(es): specify linear or non-linear and define image types used for transformation OR indicate that data were not normalized and explain rationale for lack of normalization.*

Normalization template — *Describe the template used for normalization/transformation, specifying subject space or group standardized space (e.g. original Talairach, MNI305, ICBM152) OR indicate that the data were not normalized.*

Noise and artifact removal — *Describe your procedure(s) for artifact and structured noise removal, specifying motion parameters, tissue signals and physiological signals (heart rate, respiration).*

Volume censoring — *Define your software and/or method and criteria for volume censoring, and state the extent of such censoring.*

Statistical modeling & inference

Model type and settings — *Specify type (mass univariate, multivariate, RSA, predictive, etc.) and describe essential details of the model at the first and second levels (e.g. fixed, random or mixed effects; drift or auto-correlation).*

Effect(s) tested — *Define precise effect in terms of the task or stimulus conditions instead of psychological concepts and indicate whether ANOVA or factorial designs were used.*

Specify type of analysis: ☐ Whole brain ☐ ROI-based ☐ Both

Statistic type for inference — *Specify voxel-wise or cluster-wise and report all relevant parameters for cluster-wise methods.*

(See Eklund et al. 2016)

Correction — *Describe the type of correction and how it is obtained for multiple comparisons (e.g. FWE, FDR, permutation or Monte Carlo).*

Models & analysis

n/a	Involved in the study
☐	☐ Functional and/or effective connectivity
☐	☐ Graph analysis
☐	☐ Multivariate modeling or predictive analysis

Functional and/or effective connectivity — *Report the measures of dependence used and the model details (e.g. Pearson correlation, partial correlation, mutual information).*

Graph analysis — *Report the dependent variable and connectivity measure, specifying weighted graph or binarized graph, subject- or group-level, and the global and/or node summaries used (e.g. clustering coefficient, efficiency, etc.).*

Multivariate modeling and predictive analysis — *Specify independent variables, features extraction and dimension reduction, model, training and evaluation metrics.*
